# Removal of Stomatin, a Membrane-Associated Cell Division Protein, Results in Specific Cellular Lipid Changes

**DOI:** 10.1021/jacs.2c07907

**Published:** 2022-09-22

**Authors:** Federico Donà, Cagakan Özbalci, Andrea Paquola, Federica Ferrentino, Stephen J. Terry, Elisabeth M. Storck, Gaoge Wang, Ulrike S. Eggert

**Affiliations:** † Randall Centre for Cell and Molecular Biophysics, King’s College London, London SE1 1UL, U.K.; ‡ Department of Chemistry, King’s College London, London SE1 1DB, U.K.

## Abstract

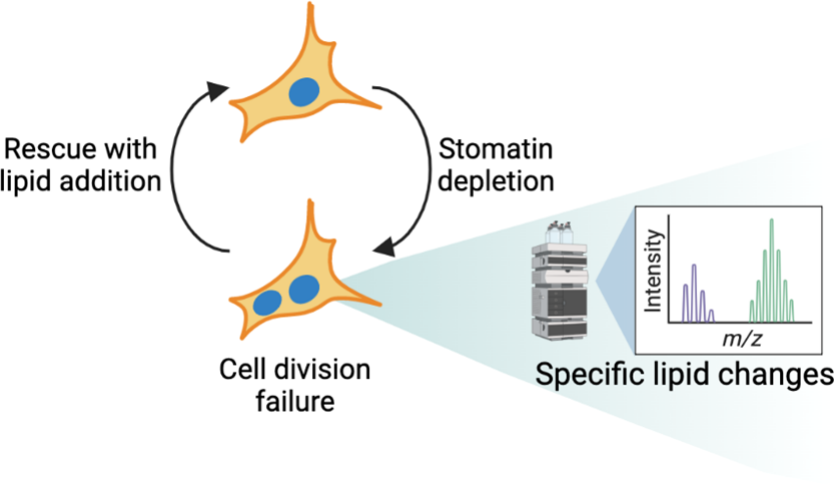

Lipids are key constituents of all cells, which express thousands of different lipid species. In most cases, it is not known why cells synthesize such diverse lipidomes, nor what regulates their metabolism. Although it is known that dividing cells specifically regulate their lipid content and that the correct lipid complement is required for successful division, it is unclear how lipids connect with the cell division machinery. Here, we report that the membrane protein stomatin is involved in the cytokinesis step of cell division. Although it is not a lipid biosynthetic enzyme, depletion of stomatin causes cells to change their lipidomes. These changes include specific lipid species, like ether lipids, and lipid families like phosphatidylcholines. Addition of exogenous phosphatidylcholines rescues stomatin-induced defects. These data suggest that stomatin interfaces with lipid metabolism. Stomatin has multiple contacts with the plasma membrane and we identify which sites are required for its role in cell division, as well as associated lipid shifts. We also show that stomatin’s mobility on the plasma membrane changes during division, further supporting the requirement for a highly regulated physical interaction between membrane lipids and this newly identified cell division protein.

## Introduction

Lipids are small molecules that are essential building blocks of cells. Together with membrane proteins, they are key constituents of all cellular membranes, including the plasma membrane and membrane-bound compartments like the endoplasmic reticulum, nuclear membrane, the Golgi complex, and mitochondria.^1^ Mammalian cells typically express thousands of chemically distinct lipids, a level of diversity approaching that of proteins. Lipids are classified into families based on their head groups, but much of lipid diversity lies in the varying length and saturation levels of their fatty acid side chains.^2^ How and why cells synthesize such diverse lipidomes is not well understood.^3^

Dividing cells ensure survival of both daughter cells by segregating their membrane-bound compartments as well as the plasma membrane, which all undergo substantial structural rearrangements during division.^4^ Work from our group and others has shown that dividing cells specifically regulate their lipid content.^5−9^ Presumably, lipids and proteins within different membrane structures work together to achieve accurate division. However, molecular details of how lipids and proteins collaborate during division are largely unknown, as are the regulatory mechanisms that drive specific lipid expression. We report here that removal of the membrane protein stomatin results in cell division failure. Although stomatin is not predicted to be involved in lipid metabolism, its depletion causes wide ranging yet specific changes in the chemical composition of the cellular lipidome, suggesting that the cell division machinery and lipid metabolism are more closely linked than has been appreciated.

## Results and Discussion

### Cells Lacking Stomatin Fail Division

**Figure 1:**
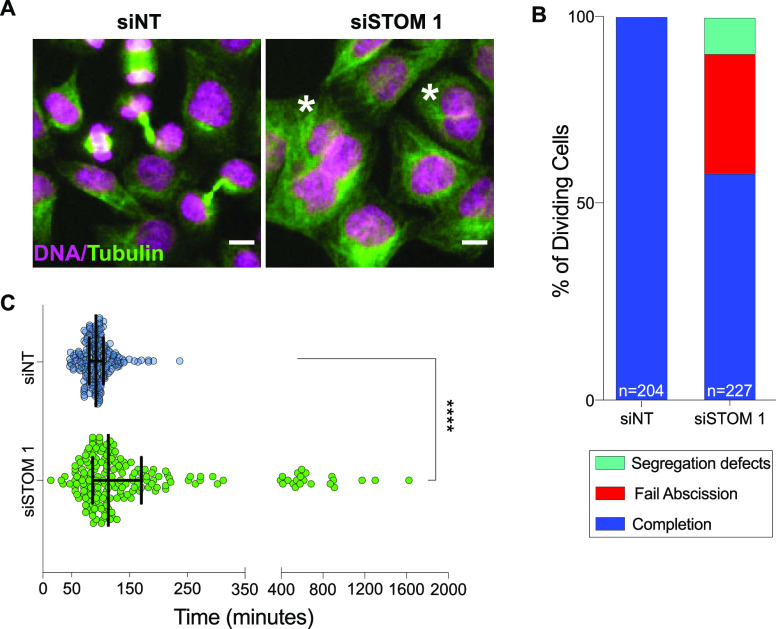
Stomatin is required for cytokinesis in human cells. (A) Representative immunofluorescence images of HeLa cells fixed and stained with anti-α-tubulin (green) and DAPI (magenta) to visualize α-tubulin and DNA, respectively, 72 h after transfection with nontargeting (siNT) or siRNA targeting stomatin (siSTOM1). * indicates bi- or multinucleated cells, scale bar = 10 μm. (B) Cytokinesis failure phenotypes for HeLa cells transfected with siSTOM1 (*n* = 227 cells) or NT siRNA (*n* = 204 cells) from three independent experiments. (C) Time taken to progress from anaphase to abscission or cytokinesis failure for siNT (blue) or siSTOM1 treated cells (green) from cells described in (B). **** indicates significance *p* < 0.0001. Black bars represent median and upper and lower quartiles.

RNA interference (RNAi) depletion of the monotopic membrane protein stomatin in HeLa cells induces a significant increase in binucleation, an indication of failed cytokinesis, the final step of division (Figures 1A and S1). Depletion of stomatin in noncancerous human corneal epithelial (HCE) cells also results in cytokinesis failure (Figure S1), showing that this protein’s function is conserved across cells derived from different tissues. As conventional CRISPR/Cas9 knockouts are difficult to amplify when cell division proteins are knocked out, we validated our RNAi results with an inducible CRISPR/Cas9 knockout of stomatin in HeLa,^10^ which also results in binucleation (Figure S1). These results confirm a role of stomatin in cytokinesis.

To determine which stage of cytokinesis is affected and to rule out increased cell fusion events, which have been reported for stomatin and can also lead to multinucleated cells,^11^ we performed time-lapse microscopy of stomatin-depleted HeLa cells expressing cell division markers GFP-tubulin and mCherry-H2B (Movies S1 and S2). We found that the predominant defect is late-stage cytokinesis failure, with a smaller percentage of cells displaying defects in chromosome segregation during mitosis (Figure 1B). Stomatin-depleted cells fail or complete cytokinesis within 117 min (median time) post-anaphase onset, whereas cytokinesis completion occurs at 96 min (median time) in control cells (Figures 1C and S1). Key cytokinesis proteins, including microtubules, actin, the septin SEPT9, the abscission protein CHMP2A (a member of the ESCRT-III complex), and the cytokinetic regulator RACGAP1 do not mis-localize in fixed cells (data not shown), suggesting that stomatin’s effects are mediated by other proteins and/or primarily due to delayed timing. Interestingly, two recent proteomic studies identified stomatin in midbodies, the site of cleavage.^12,13^ These data taken together suggest that stomatin’s primary role is during late stages of cytokinesis.

### Stomatin Depletion Causes Specific Lipid Changes

Stomatin is a ubiquitously expressed and evolutionarily conserved member of a family of membrane proteins with poorly understood functions. Loss of stomatin results in overhydrated hereditary stomatocytosis, a hemolytic anemia connected to a dysregulation of ion channels.^14^ Stomatin is linked to the membrane through an intramembrane (IM) domain, two palmitoylated cysteines, and a C-terminal region, which binds lipid rafts, likely via a cholesterol binding motif (Figure S2).^15−18^ Stomatin lacks predicted enzymatic domains and is thought to be primarily a scaffolding protein. Inspired by stomatin’s multiple membrane contacts and potential for different lipid interactions, we wondered if it could interface with determinants of lipid composition.

**Figure 2:**
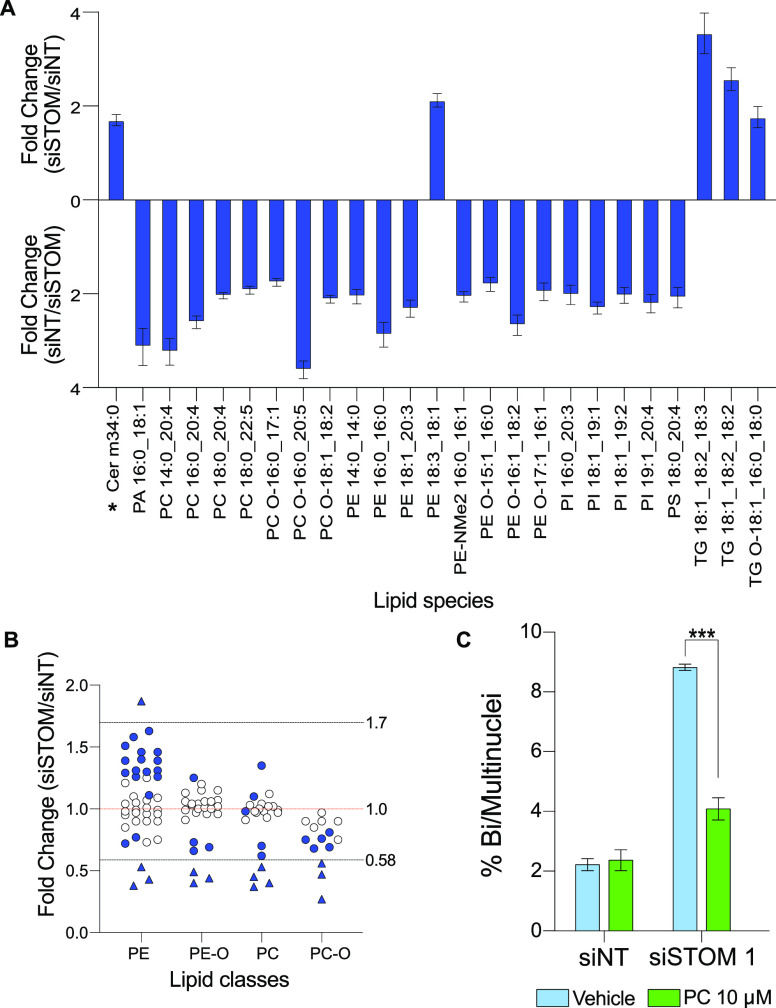
Stomatin depletion induces lipidome changes. (A) Top 25 lipid species that change in siSTOM1- vs siNT-treated cells (fold change > 1.7), sorted by lipid family. The labels on the *x* axis represent lipid names, with the letters identifying the lipid family and the numbers the length and saturation of the side chains (see structures in Figure S5). *Assignment of this species predicted by MS-FINDER but unconfirmed. Data are presented as mean ± S.D. (*N* = 9). (B) Selected lipid family changes in siSTOM vs siNT treated cells. An average of the fold changes for each lipid species within a family from all species in our in-house library are shown (lipids with a *p*-values <0.05 are shown in blue). PC is phosphatidylcholine, PC-O ether PC, PE phosphatidylethanolamine, PE-O ether PE. Triangles show lipids that appear in Figure 2A. Data from Table S2. (C) Quantification of the percentage of binucleated HeLa cells treated with siNT or siSTOM with (green) or without (light blue) addition of 10 μM PC mix. Data represent mean ± S.D. (*N* = 3), >300 cells scored per experiment, *** indicates *p* < 0.001.

We tested this hypothesis using global lipidomic analysis by liquid chromatography–mass spectrometry (LC–MS) and compared the lipidomes of cells treated with nontargeting control siRNA and cells where stomatin had been depleted by RNAi. This comparison is necessary to rule out lipid changes induced by transfection reagents.^19^ Due to the chemical diversity of lipids, fragmentation by tandem MS followed by analysis using lipidomics databases is required to identify each lipid species. To streamline this process, we created an in-house database where we have assigned by MS/MS all major lipid species from HeLa cells, as well as lower abundance species that we have associated with biological roles. Intriguingly, this analysis revealed diverse and broad changes (Figure 2) in stomatin-depleted cells compared to control, both in general lipid classes and in specific lipid species.

### Lipid Classes and Individual Lipid Species Change upon Stomatin Depletion

Using a 1.7-fold change as a cutoff for ease of analysis, we identified 25 lipids that were increased (5 species) or decreased (20 species) in stomatin-depleted cells relative to control (Figure 2A and Table S1). Three of the five species that were increased were triacylglycerols (TGs), lipids that mostly reside in lipid droplets and are primarily involved in energy storage. However, energy metabolism, measured by the cellular concentration of ATP was unaffected (Figure S3). Cells express hundreds of different TGs as they each contain three fatty acyl side chains that can all vary in length and saturation (Figure S5). Our data showing that just three species increase in response to stomatin depletion suggest that some species within this lipid family may have roles in addition to energy metabolism.

The 20 lipid species that strongly decreased in response to stomatin depletion are distributed across several major phospholipid families, including phosphatidylcholines (PCs), phosphatidylethanolamines (PEs), and phosphatidylinositols (PIs) (Figure 2A and Table S1). This diverse group highlights the biological importance of complex and cross-species lipid interactions. We also used an adapted LC–MS protocol to identify phosphorylated PIs (PIPs), which have known roles in cytokinesis. Also, enzymes modifying PIPs, such as OCRL1, are required for late stages of cytokinesis.^8,20,21^ These lipids were unaffected by stomatin depletion (Figure S4A). The group of strongly decreased lipids includes ether-linked PE (PE-O) and PC (PC-O) species (Figure S5). Ether lipids are less common than conventional ester-linked species, but are thought to have important and distinct functions, for example in cancer.^22^ It has been proposed that physical properties such as lipid packing and the area occupied by each molecule differ between ether and ester lipids, although these properties can vary according to the lipid head group and it is not clear how well these studies from reductionist model systems translate to the complex mixture of lipids and proteins in cells.^23^

Our data on single lipid species prompted us to investigate whether the cells’ response to stomatin loss resulted in systemic changes in overall lipid families or if the changes were driven by individual lipid species. Systemic changes of lipid families would likely be caused by changes in lipid biosynthetic pathways and be related to the physical properties of the lipid family, while single species changes could indicate signaling roles or specific interactions with membrane proteins. We found both: in addition to the specific species changed as discussed above (Figure 2A), some lipid families exhibited systemic changes (Figure 2B).

### Stomatin-Dependent Cell Division Failure Is Rescued by Lipid Addition

Many PCs and PC-Os were downregulated or unchanged, and the overall abundance of PC species was also decreased. In contrast, different PE species were both up- and downregulated, resulting in an overall increase of PEs (Figure 2B). We directly tested the involvement of these lipids by adding exogenous PCs to stomatin-depleted cells. As the PC species we identified (Figure 2) are not all commercially available, we used a mix of PCs isolated from bovine liver extract, with a comparable fatty acid profile. Addition of liver PCs to stomatin-depleted cells rescued cytokinesis failure, but did not impact cytokinesis in wild-type cells, showing a direct link between PC lipids, stomatin, and cytokinesis (Figure 2C). A recent study of the lipid composition the inner and outer leaflets of the plasma membrane in red blood cells showed that PEs were almost entirely found on the cytoplasmic side, while PCs were distributed across both leaflets, with the majority on the exocytic side.^24^ PCs are thought to promote flat membrane structures, whereas conical PEs support negatively curved membranes,^2^ which is the topology required for successful division. It is tempting to speculate that stomatin loss induces local changes to membrane topology, which is restored by exogenous PC addition, enabling successful division.

### Lipid Changes Are Specific to Stomatin Depletion, Not General Cell Division Failure

There was no overlap between lipid species that strongly change in response to stomatin depletion and lipids we had previously shown to accumulate in dividing cells.^7^ In addition, a lipidomic analysis of cells lacking the key cytokinesis protein CHMP4B (part of the ESCRT-III complex required for abscission), showed little overlap with stomatin-depleted cells (Figure S4B, only PE 14:0_14:0 and mCer34:0 significantly decreased/increased under both depletion conditions), suggesting that the lipid changes we observe are not due to generic cytokinesis failure. This means that normally dividing cells require a specific lipid complement, which can change in response to a negative stimulus such as stomatin depletion, possibly partly as an attempt of the cell to overcome this defect. Remarkably, stomatin does not have any predicted lipid biosynthetic activity, yet its removal profoundly impacts the cell’s lipid composition, highlighting how there is much we do not yet understand about how intricately lipids and proteins are connected.

### Specific Membrane Association Is Required for Stomatin’s Function during Division

**Figure 3:**
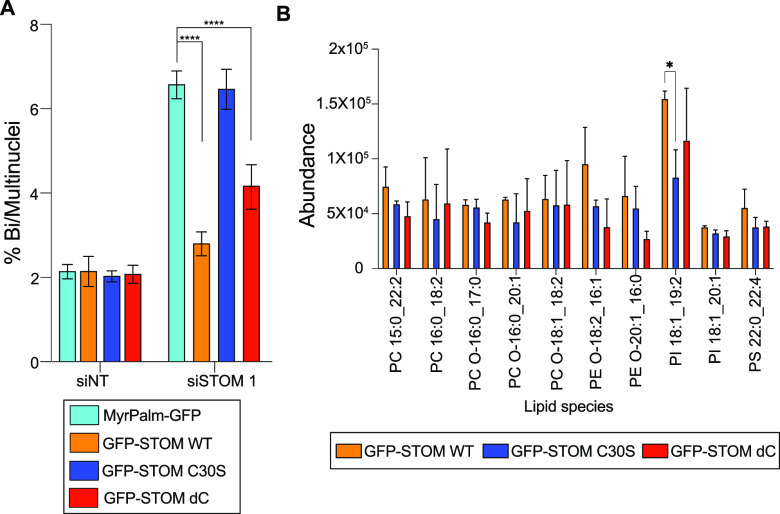
Stomatin mutants show that specific membrane association is required for cytokinesis. (A) Quantification of binucleation in HeLa stably expressing control membrane marker (MyrPalm-GFP) or GFP-tagged stomatin wt or mutant constructs, 72 h after transfection with nontargeting (NT) or siRNA targeting stomatin (siSTOM1). Data are presented as mean ± S.D. (*N* = 3), >300 cells scored per experiment, **** indicates *p* < 0.0001. (B) Lipid analysis from pulldown experiments with GFP-stomatin WT (orange), C30S (blue) or dC (red). Data are presented as mean ± S.D. (*N* = 3) *, *p* ≤ 0.05.

To understand better how stomatin may influence lipid metabolism and be involved in cell division, we created cell lines stably expressing wild-type (wt) or mutant stomatin coupled to the green fluorescent protein (GFP). We used as control a cell line stably expressing a general plasma membrane marker—myristoylated and palmitoylated GFP (MyrPalm-GFP). We focused on two mutants that would perturb stomatin’s membrane association, which we hypothesized would be required for its role in cytokinesis. Cysteine 30 resides in stomatin’s intramembrane domain and is palmitoylated, further anchoring this domain in the membrane.^15^ We mutated cysteine 30 to serine (C30S, Figure S2), which results in a loss of palmitoylation at this residue and therefore partly disrupts membrane association. We also created a construct that lacked stomatin’s C-terminal domain (ΔC (or dC in figures), Figure S2), which was shown to bind to cholesterol in vitro and is required for stomatin’s binding to lipid rafts as well as its ability to oligomerize. Both mutants have been characterized in nondividing cells.^15^ We expressed wt or mutant constructs in the absence of endogenous protein. Cells expressing wt GFP-Stomatin were able to compensate for the lack of the endogenous protein, meaning that they did not fail cytokinesis (Figure 3A). Similarly, cells expressing the ΔC construct failed cytokinesis at a significantly lower rate than control, which expressed membrane marker MyrPalm-GFP. In contrast, the C30S construct was unable to rescue cytokinesis failure, indicating that C30 palmitoylation is required for stomatin’s cytokinesis function while the C-terminal domain is not. This suggests that the association of stomatin with lipid rafts is not needed for its role in division, nor is its ability to oligomerize.

### Stomatin and Mutants Associate with Specific Lipids

To determine if stomatin wt and mutants directly bind to any lipids of interest, we performed pulldown experiments of the GFP-tagged constructs in detergent-free conditions, followed by lipid extraction. Remarkably, compared to MyrPalm-GFP, all three constructs associated with a total of 10 specific lipids (Figure 3B) and three of these lipids (PI 18:1_19:2, PE-O 16:1_18:2, PC-O 18:1_18:2) also change upon stomatin depletion (Figure 2A). These data show a clear and specific association of stomatin and its mutants with key lipid species, some of which are depleted in the absence of stomatin. Although the error in these experiments is quite large as would be expected for an indirect pulldown, we found that one of these lipid species (PI 18:1_19:2) binds less well to the inactive C30S mutant, hinting at loss of specific protein-lipid interactions in nonfunctional stomatin.

### Correlation between Stomatin Mutants, Lipid Composition and Cell Division

**Figure 4:**
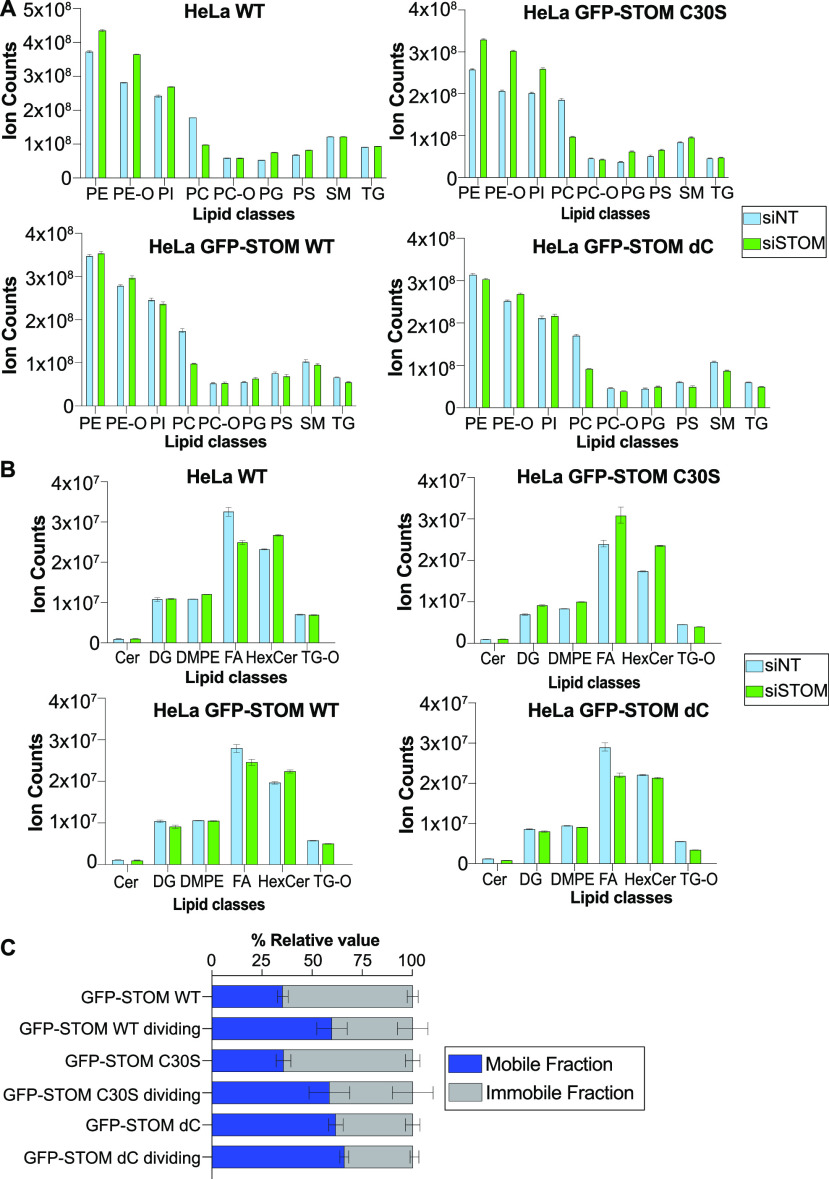
Stomatin mutants reveal links between lipid expression and protein functions. (A) and (B) Sum of the average total ion counts of each lipid class in HeLa WT or expressing different stomatin constructs treated with siNT (light blue) or siSTOM (green). HeLa and C30S fail cytokinesis after siSTOM, while GFP-Stom wt and dC do not. High abundance lipids are shown in (A), low abundance lipids are shown in (B). PC, phosphatidylcholine; PC-O, ether PC; PE, phosphatidylethanolamine; PE-O, ether PE; CER, ceramide; PG, phosphatidylglycerol; PS, phosphatidylserine; PI, phosphatidylinositol; SM, sphingomyelin; TG, triacylglycerol; TG-O, ether triacylglycerol; DG, diacylglycerol; FA, fatty acid; HexCer, hexosylceramide; and DMPE, dimethyl PE. Data are presented as sum ± S.D. (*N* = 6). (C) Distribution of mobile (gray columns) and immobile fractions (blue columns) after FRAP treatment of wt or mutant stomatin-GFP, in dividing or nondividing cells. Data are presented as mean (*N* = 2, cells = 15) and the error indicates S.E.M.

We next compared the lipidomes of the three cell lines expressing wt or mutant constructs (Figure 4A), in the presence or absence of endogenous stomatin. Further supporting our hypothesis that stomatin interfaces with lipid metabolism, we found that overexpression of all three constructs results in altered lipidomes, and these differ depending on the construct, confirming that there are multiple interactions. As this result confounds any analysis of individual lipids as shown in Figure 2A, we instead analyzed systemic lipidomic changes. Comparing lipid expression in the presence and absence of stomatin, wild-type HeLa not expressing any construct as well as HeLa expressing the C30S mutant (both fail cytokinesis when lacking stomatin) should have similar patterns. In contrast, rescue constructs wt and ΔC should have similar patterns, which should be different from the cells that have failed cytokinesis. This is what we observed (Figure 4A,B): HeLa and C30S change their lipidomes more strongly, and with the same trends, when endogenous stomatin is depleted than wt and ΔC. For example, PEs, PE-Os, and PIs are increased. PCs are decreased in all four cell lines, further highlighting the important relationship between this lipid family and stomatin. Overall, these data show that cells adapt their lipidomes more severely when they lack a version of stomatin capable of functioning during cytokinesis, supporting our hypothesis that this protein links lipid metabolism to the correct execution of cytokinesis.

### Stomatin Mobility in the Plasma Membrane Changes during Division

Both stomatin mutants and the wt protein have similar localizations in dividing cells: at the plasma membrane and at the ingressing cleavage furrow, especially at late stages of division (Figure S6 and Movie S3). Yet, the C30S mutant cannot rescue cytokinesis failure, suggesting that correct localization at the macroscopic level is insufficient for function in this situation. We therefore investigated the dynamics of the GFP-tagged mutant and wt proteins using fluorescence recovery after photobleaching (FRAP). These experiments were done in the presence of endogenous protein and therefore also report on the ability of the constructs to oligomerize with endogenous stomatin. For both wt and the C30S mutant, the proportion of the mobile fraction increased in dividing cells relative to nondividing cells (Figures 4C and S7), to a level that was comparable to that of the ΔC mutant in dividing and nondividing cells (Figures 4B and S7). These data show that a mobility change occurs during division, suggesting that stomatin uncouples from its oligomerized and lipid-raft bound state to become similar to the ΔC mutant during division. This could explain why expression of the ΔC mutant rescues cell division failure. However, this mobility change alone is not sufficient for stomatin’s role in division as the inactive C30S mutant is still able to shift its mobility pattern, showing that the anchor provided by C30 palmitoylation within the intramembrane domain is required for stomatin’s cytokinesis activity. Given the substantial lipid changes we observed upon stomatin depletion, it is tempting to speculate that this region of the protein might be involved in sensing and responding to lipid composition.

## Conclusions

We report here a new role in cell division for the integral membrane protein stomatin. We show that palmitoylation at C30, a residue within stomatin’s intramembrane domain is required for this role, while the protein’s C terminus, which mediates oligomerization and lipid-raft binding is not, suggesting that stomatin has multiple functions mediated by its different domains. Importantly, we observed that cells change their lipid composition in response to stomatin depletion, and that lipidomic changes correlate with the expression of active or inactive stomatin mutants. This was an unexpected result, as stomatin has never been implicated in lipid metabolism. Despite their essential involvement in nearly every biological and physiological process, little is known about which signals govern lipid expression patterns and even less about how these signals are relayed to the lipid biosynthetic machinery. Our data show that stomatin, directly or indirectly, can influence lipid metabolism, providing new insights into this elusive and critical question.

## Supporting Information Available

Detailed experimental methods: cell culture (including RNAi and CRISPR), microscopy and lipidomic analyses; Supporting figures: additional lipidomic data, cell data and structures of key lipids (PDF)
Top 25 changed lipids upon stomatin depletion (XLSX)
Changed lipids upon stomatin depletion (XLSX)
HeLa cell transfected with NT siRNA complete division (MOV)
Stomatin depleted HeLa cell fails cytokines after furrowing (MOV)
GFP-Stomatin WT and its mutants localize at the plasma membrane during cell division (AVI)

## Supplementary Material

ja2c07907_si_003.xlsx

ja2c07907_si_004.mov

ja2c07907_si_005.mov

ja2c07907_si_006.avi
