# Supplementary material for: Removal of Stomatin, a Membrane-Associated Cell Division Protein, Results in Specific Cellular Lipid Changes
Source: J Am Chem Soc. 2022 Sep 22;144(39):18069–74. doi: 10.1021/jacs.2c07907 (PMC9545149; doi:10.1021/jacs.2c07907)
Supplement: Supplementary file 1 — ja2c07907_si_001.pdf [file ja2c07907_si_001.pdf]

SUPPORTING INFORMATION

Removal of Stomatin, a membrane-associated cell division protein, results in specific cellular lipid changes

Federico Donà[1†], Cagakan Özbalci[1], Andrea Paquola[1,2], Federica Ferrentino[1,2], Stephen J. Terry[1§], Elisabeth M. Storck[1], Gaoge Wang[1], Ulrike S. Eggert[1,2*]

[1] Randall Centre for Cell and Molecular Biophysics, King's College London, London, SE1 1UL, UK

[2] Department of Chemistry, King's College London, London, SE1 1DB, UK

Table of contents:

1. Experimental Section 2
   1.1. Cell Culture 2
   1.2. siRNA transfections 2
   1.3. DNA constructs 2
   1.4. Production of stable cell lines 2
   1.5. CRISPR/Cas9 cell line 3
   1.6. Antibodies and fluorescent dyes 3
   1.7. Immunofluorescence and live cell imaging 3
   1.8. Western Blots 4
   1.9. Fluorescence recovery after photobleaching (FRAP) 4
   1.10. Lipidomics analyses by UHPLC-MS 5
   1.11. Lipid identification for untargeted analysis 7
   1.12. Targeted data analysis by in-house lipid library 7
   1.13. Identification of PIPs 8
   1.14. Lipid pull-down experiment 8
   1.15. ViaLight™ plus assay 9
   1.16. PC rescue experiment 9
   1.17. Statistical analysis and data presentation 9
2. Supplementary Figures 10
3. Supplementary Table and Movie Legends 19
4. Supplementary References 19

1. Experimental Section

1.1. Cell Culture

HeLa cells were verified as HeLa by STR profiling from Eurofins MWG. HeLa–mCherry-Histone, tubulin-EGFP (GTRH) were a gift from Daniel Gerlich (IMBA, Vienna). HCEs were a gift from Min S. Chang (Vanderbilt University, Nashville, Tennessee). GP-2-293 cells, utilized for retrovirus production, were from Clontech, Takara Bio, Mountain View, CA. HeLa cells stably expressing the inducible CRISPR/Cas9 plasmid[1] were a gift from Iain Cheeseman (Whitehead Institute for Biomedical Research, Cambridge, USA). All cell lines were cultured at 37°C and 5% CO_2 in DMEM (high glucose Glutamax, pyruvate) (Gibco, Life Technologies) supplemented with 10% heat inactivated fetal calf serum (Gibco, Life Technologies) and 1% Penicillin/Streptomycin (Sigma).

1.2. siRNA transfections

For RNAi experiments siRNAs were individual oligos from Qiagen (siSTOM1 and siSTOM2) or SMARTpools from Dharmacon (siCHMP4B). For siRNA transfections, Dharmafect-1 transfection reagent (Dharmacon) was used according to manufacturer's instructions using a final concentration of 40nM siRNA for 6 h. Cells were transfected the day after plating in DMEM containing no pen/strep. Samples for immunofluorescence, immunoblots and lipidomics were collected and processed 72 h, and for live cell imaging 30-34 h, after transfection. The sequences of the siRNAs oligos are listed below:

non-targeting (siNT) control (Cat. no. 1022076): UGGUUUACAUGUCGACUA

siSTOM1 (Cat no. SI04264764): ACCUAGAGAAGUCUAAGCAUA

siSTOM2 (Cat no. SI04311552): CCAGAAUGUAUAGCAGUUAUA

siCHMP4B (Cat. no. M-018075-00): GCAGAUCGACGGCACAUUA, GGACAUCGAUAAAGUUGAU, GGAAAUCAGUGGACCCGAA, GGACACGGAAGAGAUGUUA

non-targeting control (siNT) used in CHMP4B lipidomics experiment (Cat. no. D-001810-10): UGGUUUACAUGUCGACUAA, UGGUUUACAUGUUGUGUGA, UGGUUUACAUGUUUUCUGA, UGGUUUACAUGUUUUCCUA

1.3. DNA constructs

Full length Stomatin cDNA (WT) was purchased from Thermo Scientific (Clone ID: 15ACJOIP, Item No: 2015ABJG3P) and pCMS28 EGFP-Stomatin was made by cloning by digestion and ligation step from Stomatin cDNA using 5' EcoR1- and 3' Xho1 sites into pCMS28-EGFP-Puro, a viral bicistronic retroviral packaging vector encoding a puromycin resistance gene linked via an IRES.1 The other two constructs Stomatin C30S (Clone ID: 16ABPTCP, Item No: 2016AA3XKP) and ΔC (Clone ID: 15ACIRLP, Item No: 2015ABJASP), purchased from Thermo Scientific, were cloned into pCMS28-EGFP-Puro using the same strategy as for the Stomatin WT. All constructs were verified by DNA sequencing from MWG.

1.4. Production of stable cell lines

Stable cell lines were created by viral transduction using MLV-based retroviruses.[2] For retroviral transduction, constructs were all in retroviral packing vector pCMS28 EGFP IRES Puro (Stomatin WT and constructs) and co-transfected with pVSVG into GP-2-293 cells. Supernatants were collected, clarified by centrifugation (200x g, 5 min), filtered (0.45μm) and used to infect target cells in the presence of 8 μg/mL polybrene (Millipore). At

multiplicity of infection (MOI) <1 cell lines were selected with Puromycin (Life Technologies) at final concentrations of 2.0 μg/mL. Selected clonal cell lines were grown on maintenance concentration of 0.5 μg/mL Puromycin.

1.5. CRISPR/Cas9 cell line

The creation of the stable cell line was performed as described by McKinley[1] with the following optimizations. The single guide RNA (sgRNA) sequences were designed using http://crispr.mit.edu. The sgRNAs were cloned into pLentiGuide-Puro, a gift from Feng Zhang (Addgene plasmid # 52963), a two vector system characterized by a U6 promoter located before the sgRNA insertion site. To insert the single guide RNA in the plasmid, a double stranded RNA was created by annealing two oligos with complementary sequences. Each pair of oligos (1 μL each, 100μM) was phosphorylated and annealed using 0.5 μL of T4 PNK (NEB #M0201S) with 1 μL of T4 Ligase Buffer (NEB) in 10 μL final volume. The reaction was put in a thermocycler for 30 min at 30°C followed by 5 min at 95°C. Then, the tubes were allowed to cool to room temperature. The annealed oligos were diluted at 1:200 in Elution Buffer (EB, Qiagen) for the following step. 5 μg of lentiGuide-Puro was digested and dephosphorylated in a mix of 3 μL of BsmBl (NEB), 3 μL FastAP Thermosensitive Alkaline Phosphatase (Thermo Fisher Scientific), 6 μL buffer (FastAP) and 0.6 μL of 1 M DTT. The mixture was incubated at 37°C for 30 min. The digested plasmid fragments were ligated to the annealed oligo. Viral particles were produced in HEK293T cells and the transfection and virus harvest were performed as above. After the viral transduction of HeLa cells expressing CRISPR/Cas9 with the sgRNA containing plasmid and Puromycin selection, cells were tested by immunofluorescence and Western blot to analyze the level of inducible knock-out of the target protein. The cell line with the best knock-out was used for single colony selection. The selected cell lines expressing inducible CRISPR/Cas9 and sgRNA (fwd; 5'-CACCGGAGAAGCGGCACACACGGGAC-3' and rev: 5'-AAACGTCCCGTGTGTGCCGCTTCTCC-3') were used to create inducible knock-out cell lines. The expression of the CRISPR/Cas9 system is under a Tetracycline (TET) promoter that can be activated with the addition of Doxycycline Hyclate (DOX) (Sigma-Aldrich) at 1 μg/mL and incubation at 37°C for 72 h. For maximum efficiency of the inducible knock-out, the medium with DOX was changed every 24 h.

1.6. Antibodies and fluorescent dyes

Primary antibodies were used against α-Tubulin (DM1A, Sigma-Aldrich), Stomatin (sc-136502, Santa Cruz Biotechnology) and GFP (clones 7.1/13.1, Roche, 11814460001). All Alexa Fluor-488, 565, 594 and 647 secondary antibodies (Jackson ImmunoResearch) were used at a dilution of 1/500. Horseradish peroxidase (HRP) conjugated secondary antibodies were used at 1/3000 (Jackson ImmunoResearch). TRITC-phalloidin (Sigma) was used at 1 μg/mL and DAPI (New England Biolabs) was used at 3 μg/mL. For FRAP analysis, Hoechst 33342 (H3570, ThermoFisher) was used at the concentration of 10 μM to visualize DNA and recognize dividing cells.

1.7. Immunofluorescence and live cell imaging

Cells were grown on 11 mm glass coverslips, 1.5 mm thickness. Cells were fixed with 4% PFA (Alfa Aesar) in PBS, then permeabilized with 0.1% Triton X-100 in PBS for 5 min and treated sequentially with 0.1% Sodium Borohydride in PBS for 30 minutes, 1% BSA in PBS for 30 min, then stained with primary antibodies in 1% BSA in PBS for 2 h at room temperature or 4°C overnight. Secondary antibodies were applied in PBS for 1 h. Cells

were imaged using inverted Nikon Eclipse microscopes using widefield epifluorescence (Ti-E) equipped with a Cool SNAP HQ 2, DS-Fi2 Color CCD camera using 20x, 40x air and 60x oil objectives (for images in Figures 1 and S1). Confocal images were acquired at the Nikon Imaging Centre at King's College London on an inverted Confocal A1R imaging system or a Yokogawa CSU-1/Andor Neo Spinning Disc Confocal equipped using a 40x air objective (for images in Figures S1F and S6B), or 100x oil objective (for images in Figure S6A).
For live cell imaging, cells lines expressing fluorescent proteins were plated on imaging µ-slide or 24 well µ-plates (Ibidi). Cells were transfected with siRNAs and imaged 30-34h after transfection in FluoroBrite media supplemented with 10% FBS, L-glutamine and sodium pyruvate (Life Technologies). Movies were acquired on Nikon Eclipse microscopes attached to widefield epifluorescence (Ti-E) (for Movie S1 and for time-lapse in Figure S1) or Confocal A1R imaging systems with environmental chambers to maintain cells at 37°C and 5% CO_2 (for FRAP experiments in Figure S7 and Movie S2). Frames were acquired typically every 3 min unless stated otherwise. Images were processed using NIS elements software (Nikon) and adjusted for brightness using Image J or Photoshop CS5.1 (Adobe).

1.8. Western Blots

Cells were lysed in Lysis Buffer (50 mM Tris pH8, 50 mM $MgCl_2$, 150 mM NaCl), including phosphatase inhibitors (1x PhosStop, Roche) and protease inhibitors (1x Complete Protease inhibitors, Roche) by scraping with a cell scraper (for a 6-well dish, 100 µL Lysis Buffer was used). Cell lysates were homogenized by syringing multiple times through a 25-gauge syringe. 3x Sample Buffer + DTT (6% SDS, 30% glycerol, 0.003% bromophenol blue, 0.3 M DTT (freshly added), 0.2 M Tris pH 6.8) was added to cells to give a final 1x concentration and samples were then boiled (100°C) for 10 min. Proteins were resolved using SDS–PAGE followed by wet-tank transfer onto nitrocellulose membrane (0.22 µm, Biorad). Membranes were probed with the indicated antisera in 5% milk in PBS-T overnight at 4°C, prior to washing and re-incubation with secondary HRP-conjugated antibodies (Jackson ImmunoResearch).

1.9. Fluorescence recovery after photobleaching (FRAP)

HeLa cells stably expressing the different Stomatin constructs were plated in a 24 well ibidi imaging plate (µ-Plate ibidi) and incubated for 24 h. FRAP analysis was conducted using a Nikon inverted Confocal A1R imaging system using 488 nm laser for FITC excitation, in addition the microscope is equipped with a stage heater at 37°C. The acquisition was performed using a 60x oil lens objective with a frame size of 512x512 pixels. A zoom of 2.39 pixels (size: 0.19 μm) was applied. For the FRAP acquisition, a Region of Interest (ROI) of 3x3 μm was selected. The pinhole was set at 1.2 AU. For acquisition, the laser power was equally set for every specimen tested, since a variable laser power setting for could introduce unwanted bias. Some transgenic proteins were more stable than others, and hence the gain control was set appropriately. The laser power was set at 7.0, and the gain was adjusted from about 600 to 850 depending on the cell line tested. 91 images (each scan represented 3 sec elapsed time) were taken for each cell. The bleach starts after 3 scans of 1 second each.[3] The images were analyzed using FIJI software and the following pipeline was optimized following Wachsmuth's work.[4] For each image 3 different round areas (ROI) were drawn of the same size (3x3μm) of the bleach region that was used during the acquisition. The area selected was including:
BLEACH (BL): The exact region bleached by the laser.
BACKGROUND (BG): A region of only noise (outside of any target fluorescence).

REFERENCE (REF): A region of fluorescence outside of the bleached region. This was used to show bleaching from repeated imaging as result of the time lapse.

This was followed by the acquisition of the single value of the three sections for each slice of the image. After that, different corrections were applied to the obtained output:

1. Subtract Background (BG) values from Bleach (BL) and Reference (REF) to obtain corrected values (corr1). This eliminates noisy signal from the data.

BL_corr1(t) = BL(t) – BG(t)

REF_corr1(t) = REF(t) – BG(t)

2. Normalize corrected Bleach to corrected Reference.

BL_corr2(t) = BL_corr1(t) / REF_corr1(t)

3. Normalize to the mean pre-bleach intensity.

BL_corr3(t) = BL_corr2(t) / BL_corr2(pre-bleach)

At this point, the data were plotted using the Curve Fitting tool of FIJI. The fitting was based on the "Exponential Recovery" features:

$y = a \bullet (1-\exp(-b \bullet x)) + c$

a = slowly recovering fraction.

b = recovery rate

c = rapidly diffusion fraction

This information was translated as the complete recovery would be equal to:

a + c = 1

The fraction of immobile protein was:

1 – a – c

and the mobile fraction was:

a + c

1.10. Lipidomics analyses by UHPLC-MS

After the RNAi knock-down in unsynchronized HeLa, HeLa GFP-Stomatin WT, HeLa GFP-Stomatin C30S and HeLa GFP-Stomatin dC, the media was removed from each well, and cells were washed twice with cold Dulbecco's phosphate-buffered saline (DPBS). The cells were harvested in 650 µL of cold DPBS by scraping and transferred to a pre-cooled Eppendorf tube on ice. 100 µL were taken for protein concentration measurements (see below), and the rest of the cells were snap frozen in liquid nitrogen and stored at -80 °C prior to lipid extraction. For the lipid extraction all solvents and the water used were LC-MS grade. Lipid extractions were normalized according to the protein measurements. Hela cells equivalent to 60 µg protein were resuspended in 200 µL DPBS in a 1.5 mL Eppendorf tube (Safe-lock tubes, Eppendorf), followed by a 30s vortex to ensure samples were fully homogenized. The extractions were performed sequentially in 10:1 and 2:1 chloroform-methanol solutions (Fisher Bioreagents, Fisher Scientific) to extract apolar lipids (e.g. sterol, DAG, TG, PC, PE, CER, lysophosphatidylcholine, lysophosphatidylethanolamine, phosphatidylglycerol) and polar lipids (PA, LPA, PS, PI, cardiolipin, lipopolysaccharide), respectively.[5] The samples were incubated with 990 µL 10:1 chloroform-methanol solvent on a rotating wheel for 2 h at 4°C. After a brief centrifugation step (9,000 xg, 2 min, 4°C), the lower organic phase was transferred to a new Eppendorf tube and evaporated in a vacuum desiccator at 37°C. The

upper aqueous phase from the previous step was further extracted by addition of 990 µL 2:1 chloroform-methanol solution and incubation on a rotating wheel at 4°C for 1 h. The lower organic phase was collected after brief centrifugation and combined with the first extraction. The samples were vortexed for 60 sec and then evaporated under a gentle N_2 stream at 37°C. The samples were stored at -80°C until analysis. The samples were resuspended in 100 µL loading buffer (1:1:2 water:acetonitrile (ACN):isopropanol). Samples were further centrifuged at the highest speed for 4 minutes at 4°C and 90 µL of the solvent transferred to HPLC vials. 4 µL and 18 µL of the lipid suspension were injected into an HPLC column for positive and negative ESI-MS modes. Ultra-high-performance liquid chromatography-mass spectrometry (UHPLC-MS) analysis was performed on an Agilent 1290 Infinity II LC System coupled to an Agilent 6550 iFunnel Q-TOF mass spectrometer (both Agilent technologies). The chromatography protocol was optimized with minor modifications from Cajka and Fiehn.[6] Briefly, lipids were separated on an Acquity UPLC CSH C18 column (100 x 2.1 mm, 1.7 µm, 130Å) (Waters), maintained at 65°C at a flow-rate of 0.6 mL/min. The mobile phases used were (A) 60:40 (v:v) ACN/H_2O and (B) 10:90 (v:v) ACN/isopropanol. In order to increase the lipidomics coverage, solvent A and B were supplemented with 10 mM ammonium formate and 0.1% formic acid for ESI positive mode and with 10 mM ammonium acetate for ESI negative mode analysis.

UHPLC analytical gradient was set to: B from 15% to 30% (0-2 min): B from 30% to 48 % (2-2.5 min); B from 48% to 82% (2.5-11 min); B from 82% to 99% (11-11.5 min) and B kept on 99% for 4 minutes in order to wash out the non-polar compounds. In 0.5 min B returned to its initial conditions and the column was equilibrated for 3 minutes for the next run. Sample needle was washed with 100% isopropanol between injections. Samples were ordered randomly, blank extractions and pooled (QC) extractions were also added to sample sequence for further quality control steps.

Q-TOF Acquisition Parameters:

The instrument was calibrated for both polarities and analyses were performed in the extended dynamic range mode (~2 GHz) in the mass range 50 to 1700 m/z. Hexakis phosphazene and purine molecules were used as calibrants during the analyses.

Instrument Parameters:

The following parameters were used for both positive and negative mode analyses; Sheath Gas Flow 11, Sheath Gas Temp 350, Nebulizer (psig) 35, Gas Flow (l/min) 14, and Gas Temp (°C) 200.

Scan Source Parameters:

The following parameters were used for both positive and negative mode analyses:
OctopoleRFPeak 750, Skimmer1 0, Fragmentor 350, Nozzle Voltage (V) 1000 and VCap 3500.

MS/MS analysis parameters:

Previously described settings remain the same, except collision energy was adjusted to 30 eV and -35 eV for positive and negative modes. In order to improve the DDA (data–dependent analyses), background noise signals were added to the exclusion list and statistically qualified features added to inclusion lists.

Data processing and statistics for untargeted analysis

Feature selection, alignment and grouping were carried out by MassHunter Profinder Software (version B.08.00, Agilent Technologies). After importing the raw files and defining their groups "Batch Recursive Feature

Extraction for Small Molecule" option was selected. For positive mode H^+, Na^+ and NH_4^+ adducts were selected while the H^-, CH_3COO^- and $HCOO^-$ adducts were selected for negative mode. Span of the retention time (RT) was set to 0.300 min and mass tolerance was set to 20 ppm + 2 mDA at the compound binning and alignment parameters tab. Saturation threshold was set to 20%, and any feature that exceeded this limit excluded from the data set. Additionally, features originated from extraction solvents were manually removed from the data set, if the peaks are existing in blank extractions, to improve the accuracy of the statistical analyses. MassHunter Profinder Software exports "compound exchange format" (*.CEF) file for each raw file from the analyses. This file contains the m/z, RT and adduct information of the selected features and can be imported by Mass Profiler Professional (MPP) for preprocessing and statistical significance tests. No data normalization or transformation were used in this analysis. Features were filtered by a 2-step quality control system. In the first step, features which did not exist in 100% of samples in any 1 out of 2 conditions were removed. In the second step any feature with more than 30% coefficient of variation (CV) were eliminated before the significance test. Remaining features from two groups (siNT and siSTOM1) with 9 replicates (n=9) were tested by Moderated T-Test ($p<0.05$) and Benjamini-Hochberg multiple testing correction with Fold Change (FC) analysis (FC>1.7) for positive and (FC>1.7) for negative mode. Selected features were added to Q-TOF MS/MS inclusion list for further lipid identification. For HeLa cells expressing different GFP-Stomatin constructs 6 replicates (n=6) were analyzed and divided in two groups (siNT and siSTOM1) tested by Moderated T-Test ($p<0.05$) and Benjamini-Hochberg multiple testing correction with Fold Change (FC) analysis (FC>1.5) for both positive and negative mode.

1.11. Lipid identification for untargeted analysis

Fragments of the selected features were analyzed by LipidBlast,[7] MS-Dial,[6] LipidMaps,[8] Lipid Annotator[9] and MS-FINDER[10] with 5 ppm precursor ion m/z tolerance. Raw peak areas of the identified lipids were re-extracted from the data set by MassHunter Profinder's "Batch Targeted Feature Extraction" script and plotted in Excel and Prism 8 by calculated FC and standard deviations (Table S1). Lipid annotations were carried out by considering the expectations of Lipidomics Standard Initiative (LSI) (https://lipidomics-standards-initiative.org/) such as the retention time consistency and the patterns of the adducts.

For lipid species from HeLa wt, except for the DG and TG species all lipids were identified and quantified by negative mode. For lipid species from GFP-Stomatin cell lines: Cer, HexCer, CL, DMPE, FA, GM3, LPE, LPE-O, LPI, PE, PE-O, PG, PI, PI-O and PS species were identified and quantified by negative mode. The SM, CE, DG, TG and TG-O species were identified and quantified by positive mode.

1.12. Targeted data analysis by in-house lipid library

We employed our in-house lipid library as a complimentary peak selecting approach in order to investigate the lipid changes in more detail (Table S2). To create the library we generated manual iterative exclusion lists similar to the work of Koelmel et al.,[11] to increase the number of the fragmented ions. MS-DIAL[6] software with Data Dependent MS/MS option was employed for initial automatic annotation. Automatically annotated lipids were manually checked and corrected, if necessary, then curated into CSV (*.CSV) files with their unique retention times and molecular weight (Nominal MW) details for further targeted data extraction. This method allowed us to monitor over 130 lipid species from 5 lipid classes (Table S2 and Fig. 2), which did not necessarily pass the Fold Change reduction process. For the lipidomic data obtained from the cell lines expressing different GFP-Stomatin constructs we monitored 500 lipid species over 15 lipid classes (Fig. 4A). All the curated lipids were

statistically tested with the same parameters as the untargeted analysis. Significance of targeted lipids are also shown in Table S2, if the p score is calculated higher than 0.05, the lipid is labelled as N.S. (non-significant). Previously identified lipids from the untargeted analysis were added to these files if they were not previously included in the library. In targeted analysis, peak integration was confirmed manually, which caused insignificant differences between the area of the peaks in targeted and untargeted analyses, as seen in Tables S1 vs. S2.

1.13. Identification of PIPs

The PIPs were extracted using a modified version of the Folch method (CHCl3/MeOH 2:1, v/v) as described by Mucksch et al.[12] 726 µL of $CHCl_3$/MeOH/ HCl (1 M) (40:80:1, v/v/v) were added to the cell lysates. HCl allows partitioning of phosphoinositides in the organic phase. 720 µL of $CHCl_3$ was then added and the tubes were vortexed for a further 5 min. 354 µL of HCl (1 M) was added and vortexed for 2 min. The tubes were centrifuged at 1000 xg for 5 min and the lower phase, containing the phosphoinositides, was transferred to fresh Eppendorf tubes. 702 µL of $CHCl_3$/MeOH/ HCl (1.185 M) (40:80:1, v/v/v) was added, and samples were vortexed for 10 sec. Samples were centrifuged at 1000 xg for 5 min and the lower phase transferred to fresh tubes. Organic phases were evaporated on a heat block at 37°C under a constant N_2 flow. Samples were then resuspended in 90 µL of MeOH/CH_2Cl_2 (4/5, v/v). 10µL of TMS-diazomethane in hexane (2 M) (Sigma Aldrich) was added to each extract. The reaction was left to proceed for 30 min at room temperature. 10µL of glacial acetic acid was then added to quench the excess of TMS-diazomethane. A beaker with glacial acetic acid was placed in the hood to prevent inhalation of possible volatile TMS-diazomethane. This reaction releases N_2 (gas) and is vigorous if a high excess of TMS-diazomethane is neutralized. Organic phases were dried and resuspended in loading buffer. Samples were analyzed by LC-MS.

1.14. Lipid pull-down experiment

HeLa cells stably expressing the different Stomatin constructs and MyrPalm-GFP (negative control), were plated in a 10 cm dish containing ~ 4 x 10^6 cells. Cells were washed with 10 mL ice-cold DPBS and collected with 1 mL of ice-cold buffer (PIPES 20mM pH 6.8 and 1x Protease inhibitor) by scraping. Cells were sonicated with an ultrasonic probe in Eppendorf tubes on ice by alternating 20s on-cycle and 30s off-cycle for a total of 3 min. Lysates were centrifuged at slow speed (3,500 xg for 5 min). Anti-GFP magnetic agarose beads (Chromotek) were washed, before equilibration, twice with ice-cold PIPES buffer (25 µL of beads were used for each sample). Lysates were equilibrated for 1 hour at 4°C with anti-GFP beads in tube rotators. After rotation, supernatants were discarded, and beads were washed twice with 500 µL ice-cold PIPES buffer. Lipids were extracted by adding 250 µL of $CHCl_3$/MeOH (2:1, v/v) to the anti-GFP beads and by vortexing twice for 60s. The organic phase was evaporated using a heat block at 37°C and a constant nitrogen flow. After resuspension in the loading buffer (1:1:2 water:acetonitrile: isopropanol), samples were analyzed by UHPLC-MS as detailed above. Mass Hunter Profinder Software (version B 10.00) was used for feature selection and Mass Profinder Professional (MPP) was used to preprocess and analyze the data. After removing features that also appeared with MyrPalm-GFP (control), the features of interest were further analyzed by MS/MS. LIPID MAPS, MS DIAL, and MS FINDER databases were used for the in-silico analysis of the fragments.

1.15. ViaLight™ plus assay

HeLa cells were plated in 96-well white walled plates (Corning™ Costar™) and proceeded to ATP level detection 72 h after siRNA transfection (see methods above). Then, cells were cooled to room temperature and washed three times using PBS. ViaLight™ plus kit (Lonza) was used to detect the ATP levels in cells according to manufacturer's instruction. Briefly, 50 µL of cell lysis reagent was added to each well and incubated for 10 min at room temperature. After the incubation, 100 µL of pre-prepared ATP Monitoring Reagent Plus (AMR Plus) was added to cells and incubated for 2 min in the dark. The ATP level in cells was then read and recorded by plate reader ClarioStar using its luminescence detection mode.

1.16. PC rescue experiment

Cells were supplemented with bovine Liver PC (840055, Avanti Polar Lipids). The lipid mix was resuspended in methanol/water 95:5 (0.5 mg/mL), then heated and sonicated. The solution was then transferred to a glass vial and dried using a stream of nitrogen. The pellet was resuspended in a BSA solution (4 mg/mL) to make a 125 µM lipid solution and incubated for 30 minutes at 37 °C to facilitate complete solution. The PC mix was then added to cells after 24h of transfection with siRNA (siNT and siSTOM 1, see method above) and left in the media for 48h.

1.17. Statistical analysis and data presentation

For counts of multinucleated cells mean and standard deviation of the mean was calculated for N >=3 independent experiments unless otherwise stated (typically >300 cells were counted for each data point experiment unless otherwise stated). Bar graphs were drawn using GraphPad Prism. For timing throughout division experiments, Group Column Scatter plots of the data are presented showing median value, graphs were drawn using GraphPad Prism. Statistical significance was assessed using an unpaired two-tailed Student's t-test assuming equal variance.

2. Supplementary Figures

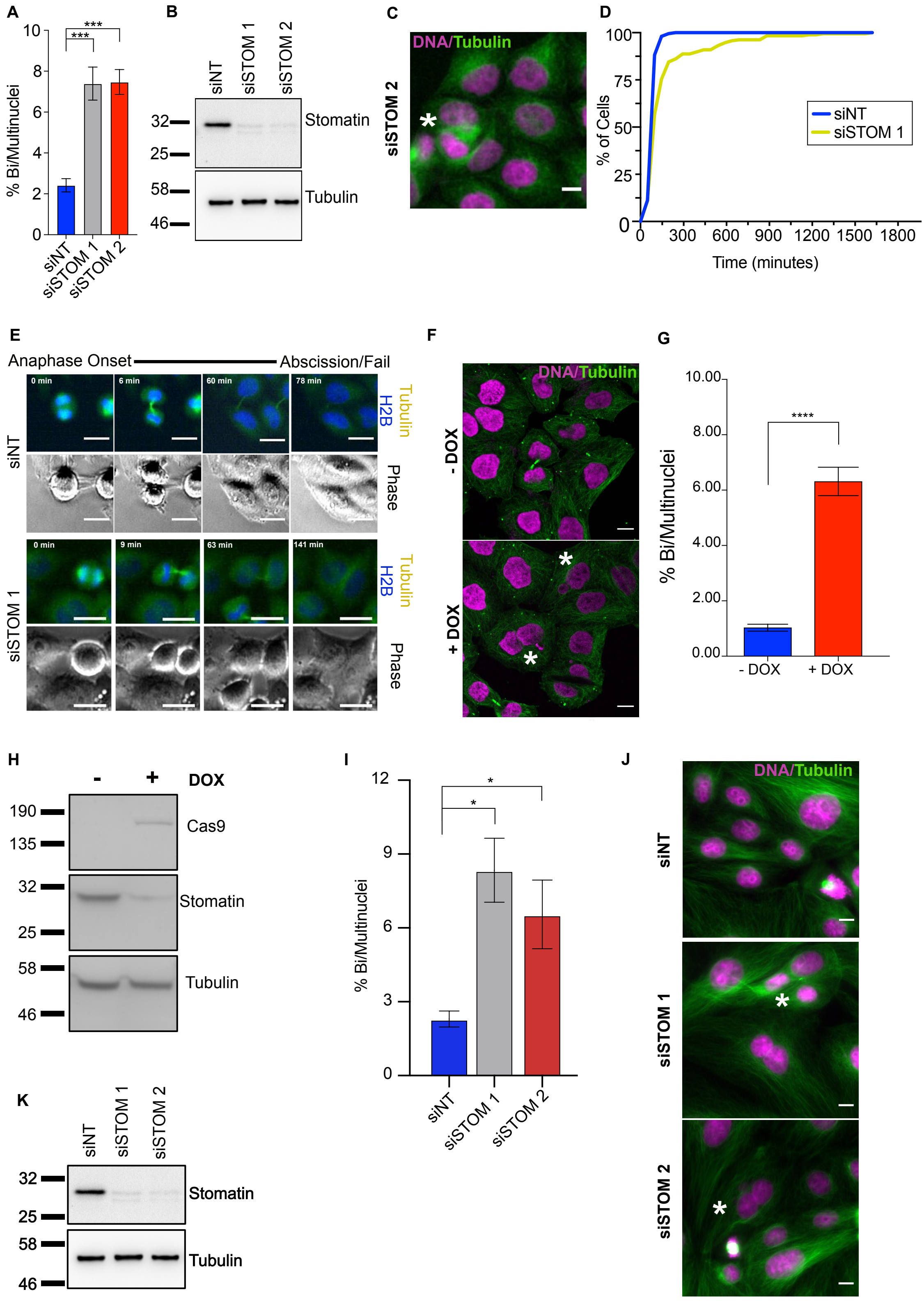

Figure S1. Stomatin is required for cytokinesis in human cells. A) Quantification of % bi/multinucleated cells after Stomatin depletion for 72 h using two individual siRNAs. Data presented as mean ± S.D. (N = 3), >300 cells scored per experiment, *** indicates $p<0.001$. B) Immunoblot showing decrease of protein levels of Stomatin following siRNA treatment in HeLa cells. α-Tubulin was used as a loading control. C) Representative immunofluorescence images of HeLa cells fixed and stained with anti-α-Tubulin (green) and DAPI (magenta) to visualize α-Tubulin and DNA, respectively, 72 h after siRNA transfection with siSTOM2. * Indicates bi- or multinucleated cells, scale bar = 10μm. D) Cumulative frequency plot of % of cells from E showing time taken to progress from anaphase to abscission. E) Stills from time-lapse movies of HeLa cell line expressing mCherry-H2B as a marker for DNA (blue) and Tubulin-GFP (green) as marker for microtubules. Cells were transfected with siNT or siSTOM1. Timings are in minutes and are adjusted to the onset of anaphase = 0 and follow cells to abscission for control or failure for Stomatin depletion. Scale bar = 20μm. F) Representative images of HeLa CRISPR/Cas9 cells fixed and stained with anti-α-Tubulin antibody (green), and DAPI (magenta) to visualize α-Tubulin and DNA, respectively, 72 h after activation of Cas9 by DOX induction. * Indicates bi- or multinucleated cells, scale bar = 10μm. G) Quantification of % bi/multinucleated cells after activation of Cas9 by DOX. Data presented as mean ± S.D. (N = 3), >300 cells scored per experiment, **** indicates $p<0.0001$ H) Immunoblot showing protein levels of endogenous Stomatin, Cas9 and α-Tubulin 72 h after activation of Cas9 by DOX. I) Quantification of J. Data presented as mean ± S.D. (N = 3), >300 cells scored per experiment, ** indicates $p<0.01$, *** indicates $p<0.001$. J) Representative images of HCE cells fixed and stained with anti-α-Tubulin (green) and DAPI (magenta) to visualize α-Tubulin and DNA, respectively, 72 h after siRNA transfection with either non-targeting control siRNA (siNT) or two independent siRNA oligos for Stomatin (siSTOM1 and siSTOM2). * Indicates bi- or multinucleated cells, scale bar = 10μm. K) Immunoblot showing decrease of protein levels of Stomatin and anti-α-Tubulin for loading control in HCE cells.

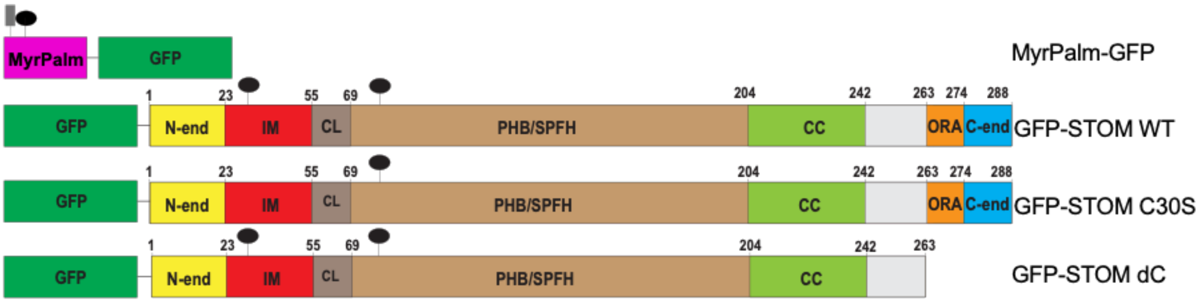

Figure S2. Schematic structures of MyrPalm-GFP, GFP-Stomatin wildtype (WT) and mutants. The different domains are highlighted: GFP is green, the MyrPalm domain is pink, Stomatin's intramembrane domain is red (IM, where 22 positively charged residues are located allowing binding to the lipid bilayer), the cholesterol recognition/interaction amino acid consensus (CRAC)-like motif (known as CL or CRAC) is grey, the prohibitin homology domain/stomatin, prohibitin, flotillin, HflK/C domain (PHB/ SPFH) is brown, the coiled-coil domain (CC) is light green, the oligomerization and lipid draft association motif (ORA) is orange and the C-terminal domain is blue, where an EA-repeat region is predicted to form a coiled-coil structure that may be key for the formation of dimers and trimers. Essential palmitoylation sites (for Stomatin and MyrPalm) are highlighted with black pins and the myristylation site is highlighted with a grey rectangular pin. Stomatin's domain structure is based on Rungaldier et al.[3]

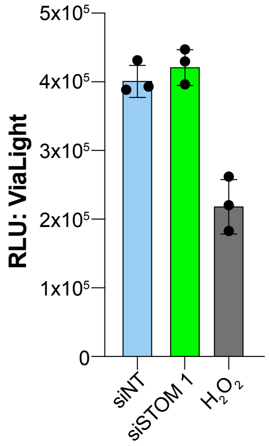

Figure S3. Depletion of Stomatin does not influence cellular metabolism. A) Bar graphs show Relative Light Units (RLU) measured by ViaLight assay in HeLa cells treated with siNT or siSTOM, H_2O_2 (9.8 mM) was used as positive control as it is known to affect mitochondrial function and therefore ATP production. Data represent mean value and standard deviation (SD), N=3.

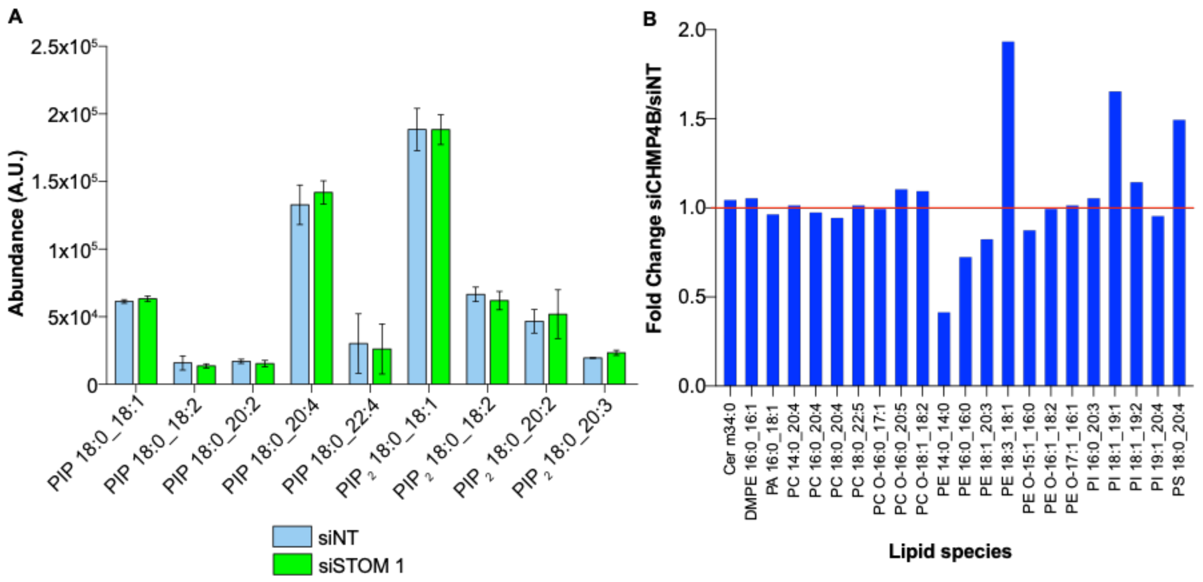

Figure S4. **A)** Stomatin depletion does not influence expression of PIPs. Bar graph showing the abundance of PIP and PIP_2 species in HeLa cells after 72h treatment with siNT or siSTOM. Note that this derivatization method followed by UHPLC-MS analysis cannot detect the position of the phosphate group(s). Data represent mean value and standard deviation (SD), (N=2). **B)** Fold change of the top 25 lipid species changed in siSTOM1 vs. siNT treated cells (Fig. 2A), in cells treated with siCHMP4B or siNT. The labels on the x axis represent lipid names, with the letters identifying the lipid family and the numbers the length and saturation of the side chains (see structures in Fig. S5). Red line shows fold change=1, where there is no difference between the two conditions. Data are presented as mean (N = 6).

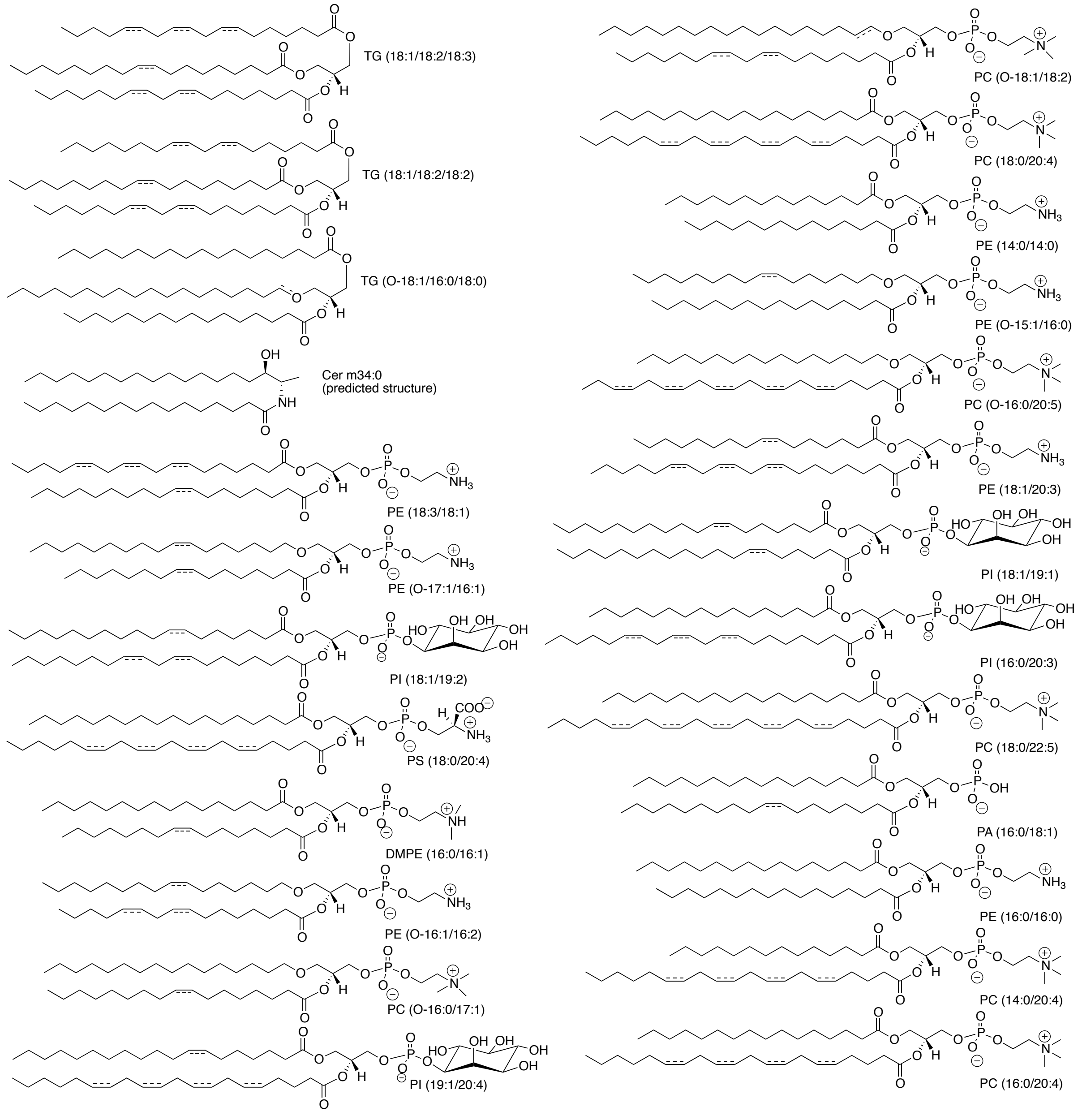

Figure S5. Likely chemical structures of 25 lipids that show the greatest change upon Stomatin depletion, as shown in Fig. 2A. Note that our MS method cannot always distinguish the position of fatty acid attachment on the glycerol backbone nor identify the locations of double bonds, including on ether lipids, where vinyl ethers (or plasmalogens) are a distinct ether lipid species. The expected protonation state at physiological pH is shown. TAG is triacylglycerol, PC is phosphatidylcholine, PE phosphatidylethanolamine, PI is phosphatidylinositol and CER ceramide.

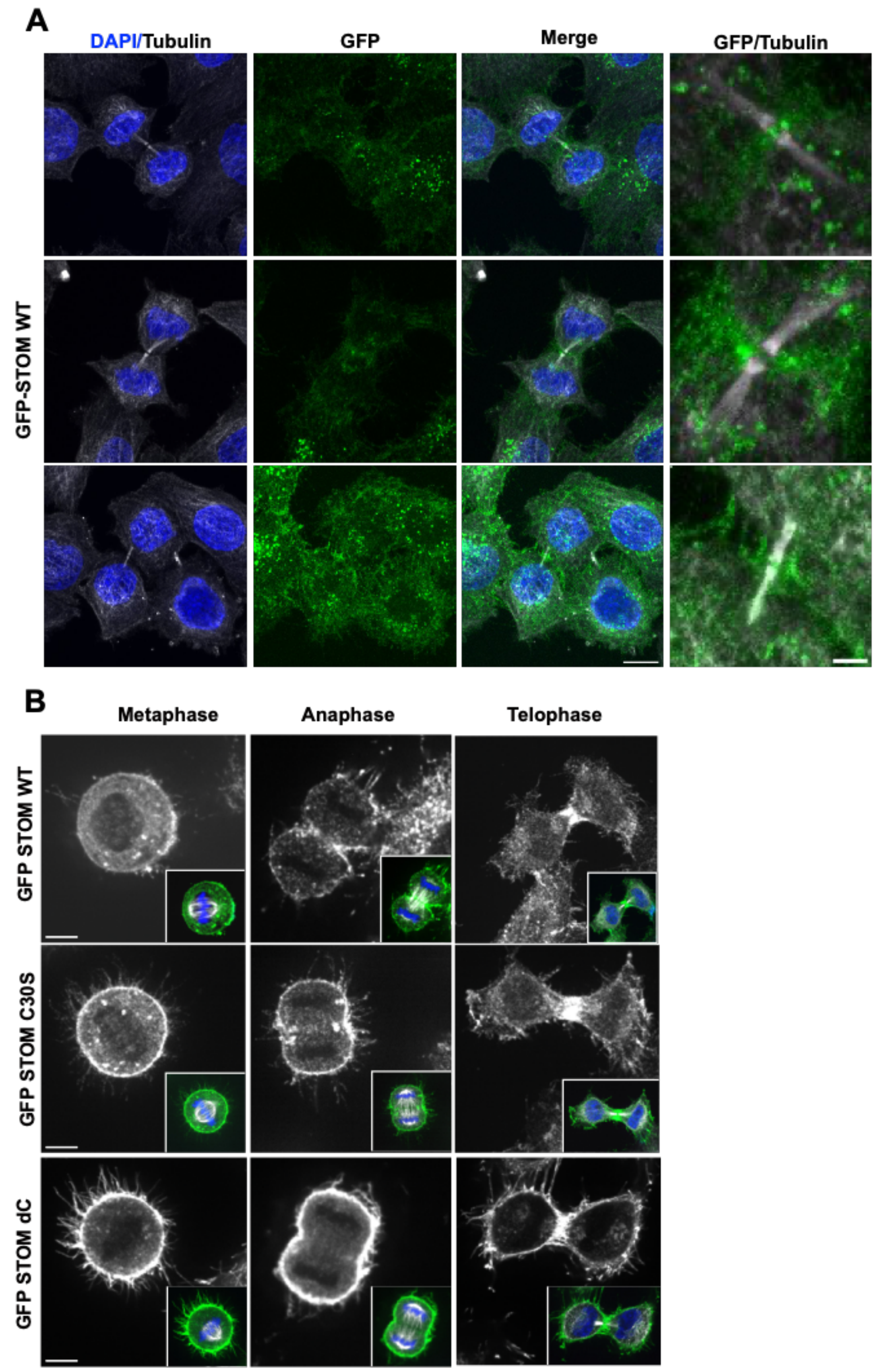

Figure S6. Stomatin and its mutants localize mostly to the plasma membrane. **A)** Representative confocal images of diving cells at late stages of cytokinesis in HeLa expressing GFP-STOM WT (green). To evaluate the correct stage of cell division cells were stained with anti- α-Tubulin (white) and DAPI (blue) to visualize α-Tubulin and DNA respectively, scale bar = 10μm. The last column shows a zoom of the midbody area to highlight the localization of GFP-STOM (green) together with α-Tubulin (white), scale bar = 2μm **B)** Representative confocal images of different stages of cell division (metaphase, anaphase and telophase/cytokinesis) in HeLa expressing GFP-STOM WT, C30S or dC, constructs (shown in white). To get a sense of the stage of cell division, the inserts

show the same cells stained with anti- α-Tubulin (white), anti-GFP (green) and DAPI (blue) to visualize α-Tubulin, Stomatin constructs and DNA respectively, scale bar = 10μm.

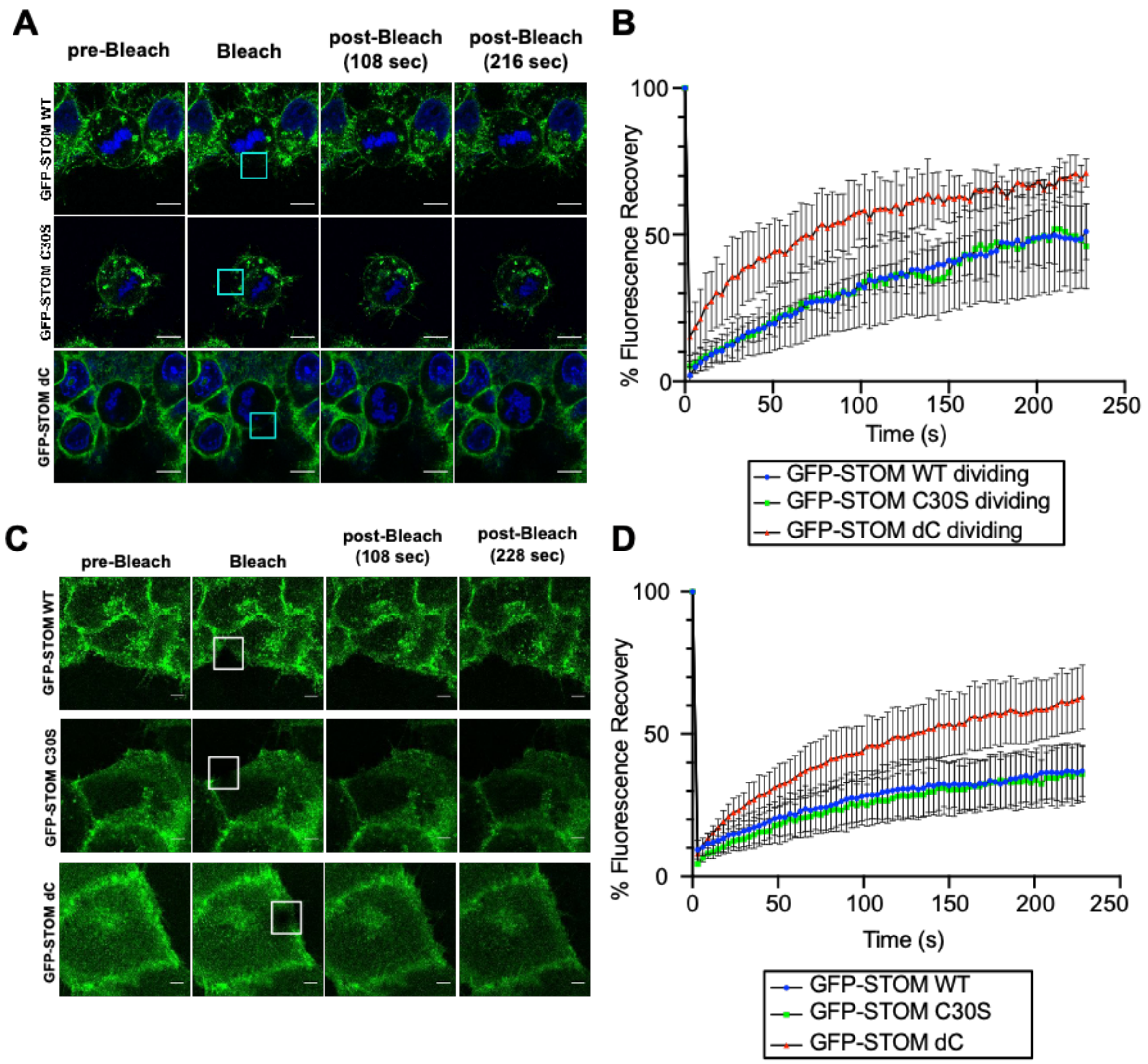

Figure S7. Fluorescence recovery after photobleaching (FRAP) analysis of HeLa GFP-Stomatin WT and mutants in dividing and non-dividing cells. A) Stills from the live imaging of FRAP of dividing HeLa cells expressing GFP-Stomatin WT, C30S or dC subjected to photobleaching 24 h post seeding. Cells were stained with cell permeable Hoechst 33342 (blue) to visualize DNA. Three images were acquired before bleaching. An area on the plasma membrane (white square in the “Bleach” column) was bleached with a 488 nm laser and the fluorescence recovery of the bleached spot was measured every 3 sec for 2.30 min. Scale bar = 20 µm. B) The graph represents the normalized fluorescence recovery in the ROI plotted against time (N = 2, cells = 10). The error indicates S.E.M. of the mean for each fitted curve. C) Stills from the live imaging of FRAP analysis of non-dividing HeLa cells expressing GFP-Stomatin WT, C30S or dC subjected to photobleaching 24 h post seeding (as in A). Scale bar = 10 µm. D) The graph represents the normalized fluorescence recovery in the ROI plotted against time (N = 2, cells = 15). The error indicates S.E.M. of the mean for each fitted curve.

3. Supplementary Table and Movie Legends

Table S1: Top 25 changed lipids upon Stomatin depletion.

Top 25 changed lipids are shown. The table shows the basic features including compound name, formula, retention time (RT), nominal mass (theory), nominal mass (measured), error (ppm), ion species, identification level, annotation software, average value of ion counts (with S.D. n=9) of NT siRNA and siSTOM treated cells, statistical significance (shown as p-value), fold change of Stomatin depleted cells over control (n=9) and S.D. for each lipid and also their annotations.

Table S2: Changed lipids upon Stomatin depletion

The table shows the changes in all identified lipids in Stomatin depleted compared to control cells, using our in-house lipid annotation library. In the table are reported the basic features including compound name, average value of ion counts (with S.D. n=9) of NT siRNA and siSTOM treated cells, statistical significance (shown as p-value), fold change of Stomatin depleted cells over control and S.D. These data are represented in Fig. 2B.

Movie S1. HeLa cell transfected with NT siRNA complete division.

Epifluorescence movie of dividing HeLa cell stably expressing GFP-tubulin (green) and H2B-mCherry (blue) transfected with control non-targeting siRNA and imaged 30 h after transfection. The corresponding brightfield movie is shown on the left. Frames were acquired every 3 minutes, playback speed is 10 frames/second, scale bar = 20μm.

Movie S2. Stomatin depleted HeLa cell fails cytokines after furrowing.

Epifluorescence movie of dividing HeLa cell stably expressing GFP-tubulin (green) and H2B-mCherry (blue) transfected with siSTOM1 and imaged 30 h after transfection. The corresponding brightfield movie is shown on the left. Frames were acquired every 3 minutes, playback speed is 10 frames/second, scale bar = 20μm.

Movie S3. GFP-Stomatin WT and its mutants localize at the plasma membrane during cell division.

HeLa cells stably expressing GFP-Stomatin WT, C30S or dC (respectively left, center and right) imaged after 24 h seeding. Frames were acquired every 5 minutes, playback speed is 10 frames/second, scale bar = 10μm.

4. Supplementary References

1. McKinley, K. L.; Cheeseman, I. M., Large-Scale Analysis of CRISPR/Cas9 Cell-Cycle Knockouts Reveals the Diversity of p53-Dependent Responses to Cell-Cycle Defects. *Dev Cell* **2017,** *40* (4), 405-420 e2.

2. Terry, S. J.; Dona, F.; Osenberg, P.; Carlton, J. G.; Eggert, U. S., Capping protein regulates actin dynamics during cytokinetic midbody maturation. *Proc Natl Acad Sci U S A* **2018,** *115* (9), 2138-2143.

3. Rungaldier, S.; Umlauf, E.; Mairhofer, M.; Salzer, U.; Thiele, C.; Prohaska, R., Structure-function analysis of human stomatin: A mutation study. *PLoS ONE* **2017**.

4. Wachsmuth, M., Molecular diffusion and binding analyzed with FRAP. *Protoplasma* **2014,** *251* (2), 373-82.

5. Sampaio, J. L.; Gerl, M. J.; Klose, C.; Ejsing, C. S.; Beug, H.; Simons, K.; Shevchenko, A., Membrane lipidome of an epithelial cell line. *Proc Natl Acad Sci U S A* **2011,** *108* (5), 1903-7.

6. Tsugawa, H.; Cajka, T.; Kind, T.; Ma, Y.; Higgins, B.; Ikeda, K.; Kanazawa, M.; VanderGheynst, J.; Fiehn, O.; Arita, M., MS-DIAL: data-independent MS/MS deconvolution for comprehensive metabolome analysis. *Nat Methods* **2015,** *12* (6), 523-6.

7. Kind, T.; Liu, K. H.; Lee, D. Y.; DeFelice, B.; Meissen, J. K.; Fiehn, O., LipidBlast in silico tandem mass spectrometry database for lipid identification. *Nat Methods* **2013,** *10* (8), 755-8.

8. O'Donnell, V. B.; Dennis, E. A.; Wakelam, M. J. O.; Subramaniam, S., LIPID MAPS: Serving the next generation of lipid researchers with tools, resources, data, and training. *Sci Signal* **2019,** *12* (563).

9. Koelmel, J. P.; Li, X.; Stow, S. M.; Sartain, M. J.; Murali, A.; Kemperman, R.; Tsugawa, H.; Takahashi, M.; Vasiliou, V.; Bowden, J. A.; Yost, R. A.; Garrett, T. J.; Kitagawa, N., Lipid Annotator: Towards Accurate Annotation in Non-Targeted Liquid Chromatography High-Resolution Tandem Mass Spectrometry (LC-HRMS/MS) Lipidomics Using A Rapid and User-Friendly Software. *Metabolites* **2020,** *10* (3).

10. Lai, Z.; Tsugawa, H.; Wohlgemuth, G.; Mehta, S.; Mueller, M.; Zheng, Y.; Ogiwara, A.; Meissen, J.; Showalter, M.; Takeuchi, K.; Kind, T.; Beal, P.; Arita, M.; Fiehn, O., Identifying metabolites by integrating metabolome databases with mass spectrometry cheminformatics. *Nature Methods* **2018,** *15* (1), 53-56.

11. Koelmel, J. P.; Kroeger, N. M.; Gill, E. L.; Ulmer, C. Z.; Bowden, J. A.; Patterson, R. E.; Yost, R. A.; Garrett, T. J., Expanding Lipidome Coverage Using LC-MS/MS Data-Dependent Acquisition with Automated Exclusion List Generation. *J Am Soc Mass Spectrom* **2017,** *28* (5), 908-917.

12. Mucksch, F.; Citir, M.; Luchtenborg, C.; Glass, B.; Traynor-Kaplan, A.; Schultz, C.; Brugger, B.; Krausslich, H. G., Quantification of phosphoinositides reveals strong enrichment of PIP2 in HIV-1 compared to producer cell membranes. *Sci Rep* **2019,** *9* (1), 17661.
